# Effects of symmetry breaking of the structurally-disordered Hamiltonian ensembles on the anisotropic decoherence of qubits

**DOI:** 10.1038/s41598-022-06891-4

**Published:** 2022-02-21

**Authors:** Hong-Bin Chen

**Affiliations:** 1 Department of Engineering Science, National Cheng Kung University, Tainan, 701401 Taiwan; 2 Center for Quantum Frontiers of Research & Technology, NCKU, Tainan, 701401 Taiwan

**Keywords:** Quantum physics, Quantum information, Quantum mechanics, Quantum simulation, Qubits, Theoretical physics

## Abstract

It is commonly known that the dephasing in open quantum systems is due to the establishment of bipartite correlations with ambient environments, which are typically difficult to be fully characterized. Recently, a new approach of average over disordered Hamiltonian ensemble is developed and shown to be capable of describing the nonclassicality of incoherent dynamics based on inferring the nonclassical nature of the correlations. Here we further extend the approach of Hamiltonian ensemble in the canonical form to the realm of structural disorder. Under the variable separation of the probability distribution within the Hamiltonian ensemble, the geometrical structure is easily visualized and can be characterized according to the degree of symmetry. We demonstrate four degrees and investigate the effects of different types of symmetry breaking on the incoherent dynamics. We show that these effects are easily understood from the emergences of additional terms in the master equations, leading to rather general master equations and, consequently, going beyond the previous frameworks of pure dephasing or isotropic depolarization.

## Introduction

Exposed to the inevitable interactions with the huge surrounding environments, any quantum systems generically undergo incoherent dynamical processes and gradually lose their quantumness^1–5^, constituting the primary obstacle in the developments of frontier quantum technologies^6–14^. Therefore, it is crucial to characterize, control, and eliminate the sources of decoherence. One of the main causes of the incoherent dynamical nature arises from the damage to the system-environment correlations established during their interactions. However, due to the huge environmental degree of freedom, it is infeasible to fully access these bipartite correlations. This renders a general, and precise, description of how the correlations are destroyed highly nontrivial. Consequently, their incoherent effects on the reduced system dynamics are taken into account in terms of a family of completely positive and trace-preserving (CPTP) dynamical linear maps^15–18^. Moreover, there are several alternative techniques for characterizing CPTP maps have been developed, such as the Kraus operators^19^, the process matrices^20^, and the Choi-Jamiołkowski isomorphism^21,22^.

Besides the system-environment interactions, incoherent dynamics can also arise from a completely different mechanism. Recently, a promising approach, referred to as Hamiltonian ensemble (HE), has been developed^23,24^. HE is initially dedicated to the investigation of disordered systems and classical noises^25–28^, which are described by an ensemble of Hermitian operators parameterized by some random variables obeying a specified probability distribution. Irrespective of the unitarity of a single realization generated by each member Hermitian operator, the time-evolved state ${\overline{\rho }}(t)$ undergoes a dephasing dynamics after the ensemble-averaging procedure over all unitary realizations^23,24^. Furthermore, it has been pointed out that the incoherent dynamical behavior is intimately related to the properties of the probability distribution^23^. Based on this insight, the probability distribution encapsulated within the HE has attracted exclusive focus in the characterization of dephasing dynamics, and been promoted to be the canonical Hamiltonian ensemble representation (CHER) of dynamical processes in the frequency domain^29,30^. Additionally, the CHER has been shown to be a versatile approach in the quantification of process nonclassicality^29^.

However, due to the difficulty imposed by the non-abelian algebraic structure underlying the canonical HE, most of the aforementioned works relied on significant simplifications to circumvent it. For example, the spectral disorder appealed to HEs consisting of diagonal operators, leading to the pure dephasing^23^. The process nonclassicality was exemplified^24^ and quantified^29^ under the same framework of Cartan subalgebra. Furthermore, the attempt going beyond the framework of pure dephasing was the unitarily invariant disorder, which was studied by incorporating the spectral disorder with the Haar measure integral, leading to the isotropic depolarization^23^. Whereas, the most drawback of this unitary invariance approach lies in the deviation from the canonical form, giving rise to the issue of double counting of each member Hermitian operator. It is also worth noting that there are efforts devoting to the decomposition of pure dephasing into random unitary (RU) representation with static probability distribution^31^. However, the generator is still time-dependent, rather than the canonical form.

To cure this issue, as well as to go further beyond the above two frameworks of pure dephasing and isotropic depolarization, here we study the canonical HE of structural disorder along with different degrees of symmetry. This not only enables us to explore more general types of qubit dephasing dynamics in terms of HE in the canonical form, but also demonstrates the effects of symmetry breaking of the geometrical structure on the qubit incoherent dynamics. The unitarily invariant disorder is shown to be a special case of the spherical symmetry. We have reduced the continuous spherical symmetry to three lower levels, until the discrete one of simultaneous reflectional symmetries. Each symmetry breaking gives rise to an additional disturbance complicating the dynamics, including the anisotropic decay rates, the effective level spacing, and the off-diagonal decay rates. Following this line, the pattern of any further generalization can be deduced. Finally, we stress that to understand attainable qubit dynamics is important, particulary when we try to find the right type of HE for a physical process.

## Hamiltonian ensemble in the spherical coordinates

We begin with presenting the general HE in the canonical form and exploring the corresponding ensemble-averaged dynamics for a qubit. Generically, any Hermitian operators acting on a qubit system are elements in the ${\mathfrak {u}}(2)$ Lie algebra of the form ${\widehat{H}}_{\vec {\lambda }}=(\lambda _0{\widehat{I}}+\lambda _x{\hat{\sigma }}_x+\lambda _y{\hat{\sigma }}_y+\lambda _z{\hat{\sigma }}_z)/2$. However, as $\lambda _0$ plays no role in describing the qubit dynamics due to the commutativity $[{\widehat{I}},{\hat{\sigma }}_j]=0$, we can restrict ourselves to the traceless operators in the ${\mathfrak {su}}(2)$ Lie algebra with $\lambda _0=0$.

Here we consider the Hamiltonian ensemble $\{(p_{\vec {\lambda }},{\widehat{H}}_{\vec {\lambda }})\}_{\vec {\lambda }}$ of canonical form parameterized by $\vec {\lambda }\in {\mathbb {R}}^3$, where each member Hamiltonian ${\widehat{H}}_{\vec {\lambda }}\in {\mathfrak {su}}(2)$ is associated with a probability $p_{\vec {\lambda }}$ of occurrence. Since the ${\mathfrak {su}}(2)$ Lie algebra is non-abelian, the corresponding unitary time-evolution operator ${\widehat{U}}_{\vec {\lambda }}=\exp (-i{\widehat{H}}_{\vec {\lambda }}t)$ is rather difficult to be dealt with in these parameters. Crucially, along with the change of variables to the spherical coordinates $\lambda _x=\omega \sin \theta \cos \phi$, $\lambda _y=\omega \sin \theta \sin \phi$, and $\lambda _z=\omega \cos \theta$, the HE can be recast into

1 $$\begin{aligned} \left\{ \left( p(\omega ,\theta ,\phi ),\frac{\omega }{2}\vec {n}\cdot {\hat{\sigma }}\right) \right\} _{(\omega ,\theta ,\phi )}, \end{aligned}$$

where $\vec {n}=(\sin \theta \cos \phi ,\sin \theta \sin \phi ,\cos \theta )\in {\mathbb {R}}^3$ is the directional unit vector and ${\hat{\sigma }}$ denotes the three Pauli matrices. Accordingly, each operator ${\widehat{U}}_{\vec {\lambda }}=\cos (\omega t/2){\widehat{I}}-i\sin (\omega t/2)\vec {n}\cdot {\hat{\sigma }}$ is explicitly expressed in the spherical coordinates and leads to a unitarily-evolved single realization

2 $$\begin{aligned} \rho _{\vec {\lambda }}(t)={\widehat{U}}_{\vec {\lambda }}\rho _0{\widehat{U}}_{\vec {\lambda }}^\dagger =\cos ^2\frac{\omega t}{2}\rho _0-i\sin \frac{\omega t}{2}\cos \frac{\omega t}{2}\left[ \vec {n}\cdot {\hat{\sigma }},\rho _0\right] +\sin ^2\frac{\omega t}{2}(\vec {n}\cdot {\hat{\sigma }})\rho _0(\vec {n}\cdot {\hat{\sigma }}), \end{aligned}$$

provided an initial state $\rho _0$.

In view of the member Hamiltonian encapsulated within the HE (1), as well as the single realization (2), we can observe a separation between the radial coordinate, $\omega$, and the solid angular coordinates, $(\theta ,\phi )$. Accordingly, we will further assume a separable probability distribution

3 $$\begin{aligned} p(\omega ,\theta ,\phi )=P(\omega )\Theta (\theta ,\phi ) \end{aligned}$$

into two positive real functions $P(\omega )$ and $\Theta (\theta ,\phi )$. We will show that many interesting properties can be conveniently studied with the help of this separation; particularly, the symmetry is easily exhibited by the geometrical structure of $\Theta (\theta ,\phi )$. One should note that, it is $p_{\vec {\lambda }}$ a legitimate probability distribution and normalized to unity $\int p_{\vec {\lambda }}d^3\vec {\lambda }=\int \int p(\omega ,\theta ,\phi )\omega ^2d\omega d\Omega =1$ with $d\Omega =\sin \theta d\theta d\phi$; while this is not the case for $P(\omega )$ or $\Theta (\theta ,\phi )$ individually. We therefore assume that $\int _0^\infty P(\omega )\omega ^2 d\omega =1/\xi$ and $\int \Theta (\theta ,\phi )d\Omega =\xi$ due to the separability. This guarantees the normalization condition for $p(\omega ,\theta ,\phi )$.

Based on the separability, the ensemble-averaged dynamics under the structurally-disordered HE (1) is given by

4 $$\begin{aligned} {\overline{\rho }}(t) & =  {} {\mathscr {E}}_t\{\rho _0\}=\int p_{\vec {\lambda }}{\widehat{U}}_{\vec {\lambda }}\rho _0{\widehat{U}}_{\vec {\lambda }}^\dagger d^3\vec {\lambda } \nonumber \\ & =  {} \frac{1}{2}\left( 1+\langle \cos \omega t\rangle _P\xi \right) \rho _0 -i\frac{1}{2}\langle \sin \omega t\rangle _P\sum _{j=1}^3\left[ \langle n_j\rangle _\Theta {\hat{\sigma }}_j,\rho _0\right] +\frac{1}{2}\left( \frac{1}{\xi }-\langle \cos \omega t\rangle _P\right) \sum _{j=1}^3\langle n_j^2 \rangle _\Theta {\hat{\sigma }}_j\rho _0{\hat{\sigma }}_j, \end{aligned}$$

where, for convenience, we have introduced the radial expectation $\langle f(\omega )\rangle _P=\int _0^\infty f(\omega )P(\omega )\omega ^2d\omega$ with respect to $P(\omega )$, $n_j$ the three components of $\vec {n}$, the 1st directional moment $\langle n_j\rangle _\Theta =\int n_j\Theta (\theta ,\phi )d\Omega$, and the 2nd directional moment $\langle n_jn_k\rangle _\Theta =\int n_jn_k\Theta (\theta ,\phi )d\Omega$. It should be noted that, in the last term of Eq. (4), there are only three square terms of the 2nd directional moment left, due to an appropriate orthogonal transformation. This means that, for any given HE configuration admitting variable separation (3), one is possible to redefine a new set of axes of the geometry by using a basis transformation to eliminate the crossing terms $\langle n_j n_k \rangle _\Theta$ with $j\ne k$. Consequently, without loss of generality, we can start from the dynamical linear map (4) and ignore the crossing terms in the following. This significantly simplifies the complexity of the problem. Further details on the orthogonal transformation are discussed in Methods.

On the other hand, the density matrix for a qubit system $\rho =({\widehat{I}}+\vec {\rho }\cdot {\hat{\sigma }})/2$ is also an element in the ${\mathfrak {u}}(2)$ Lie algebra parameterized by the corresponding Bloch vector $\vec {\rho }\in {\mathbb {R}}^3$. Therefore, the properties of the dynamical linear map (4) can be fully understood from its action on the generators of the ${\mathfrak {u}}(2)$ Lie algebra:

5 $$\begin{aligned} \left\{ \begin{array}{l} {\mathscr {E}}_t\{{\widehat{I}}\}={\widehat{I}} \\ {\mathscr {E}}_t\{{\hat{\sigma }}_j\}=f_j(t){\hat{\sigma }}_j +\sum _{k,l=1}^{3}\varepsilon _{jkl}\langle \sin \omega t\rangle _P\langle n_l\rangle _\Theta {\hat{\sigma }}_k, j=1,2,3 \end{array}\right., \end{aligned}$$

where

6 $$\begin{aligned} f_j(t)=\langle \cos \omega t\rangle _P\left( \xi -\langle n_j^2 \rangle _\Theta \right) +\langle n_j^2 \rangle _\Theta /\xi , \end{aligned}$$

and $\varepsilon _{jkl}=1$ if (*j*, *k*, *l*) is an even permutation of (1, 2, 3), $-1$ if an odd permutation, and 0 otherwise. From the first line of Eq. (5), we can see that ${\mathscr {E}}_t$ is unital. This can be understood if we note that the HE is a special case with time-independent probability distribution and member Hamiltonian of a superset of RU. Due to the unitality, the decohering behavior of ${\mathscr {E}}_t$ is of the type of depolarization, captured by the second line of Eq. (5). From the later, we can see that the decohering behavior of ${\mathscr {E}}_t$ depends highly on the structure and the symmetry of $p(\omega ,\theta ,\phi )$ via the radial expectations and the directional moments. On the other hand, this also reflects the notion of CHER^29,30^, which conceives the (quasi-)distribution function as the characteristic representation of a dynamics.

Accordingly, we will investigate the dynamical behavior of ${\mathscr {E}}_t$ along with different degrees of symmetry of the probability distribution exhibited by the solid angular part $\Theta (\theta ,\phi )$.

## Spherical symmetry

**Figure 1:**
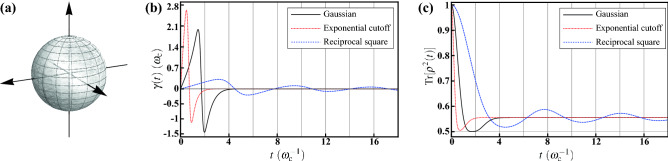
Decoherence under the HE with spherically symmetric probability distribution. (**a**) Visualization of the solid angular part $\Theta (\theta ,\phi )=1/4\pi$ of the probability distribution. As $\Theta (\theta ,\phi )$ is a constant, the geometry is a sphere of radius $1/4\pi$. (**b**) Time-evolution of the decay rates $\gamma (t)$ for the Gaussian (black solid curve), the exponential cutoff (red dotted curve), and the reciprocal square (blue dashed cruve) radial functions. Their behaviors are different to each other, as explained in the main text. The decay rates temporarily go down to negative values in some time periods, indicating transitions between indivisibility and full divisibility of the type of random unitary. (**c**) The purities $\mathrm {Tr}[{\overline{\rho }}^2(t)]$ governed by the isotropic depolarization with pure initial states for the three radial functions. There is a rivival in the time period of negative decay rate. Additionally, the final value of the purities is 5/9, rather than 1/2, reflecting that the final state is not the completely mixed state.

We first consider the case of spherically symmetric probability distribution with $\Theta (\theta ,\phi )=1/4\pi$. Fig. 1a shows its visualization with the distance between the surface and the origin indicating the value of $\Theta (\theta ,\phi )$ in solid angular coordinates $(\theta ,\phi )$. As $\Theta (\theta ,\phi )$ is a constant, the geometry forms a sphere; therefore, the HE is of spherical symmetry, which is the highest symmetry can be exhibited. It is straightforward to verify that $\int _0^\infty P(\omega )\omega ^2 d\omega =\int \Theta (\theta ,\phi )d\Omega =1$, the 1st directional moments $\langle n_j\rangle _\Theta =0$, and the 2nd directional moments $\langle n_j n_k\rangle _\Theta =\delta _{jk}/3$, satisfying the diagonal condition of Eq. (4).

Under such highly symmetric geometry, from Eq. (5), the ensemble-averaged dynamics is given by

7 $$\begin{aligned} {\overline{\rho }}(t)={\mathscr {E}}_t\{\rho _0\}=\left[ 1-w(t)\right] \frac{{\widehat{I}}}{2}+w(t)\rho _0, \end{aligned}$$

which is a statistical mixture between the completely mixed state and the initial state $\rho _0$ along with a time-varying wight $w(t)=(2\langle \cos \omega t\rangle _P+1)/3$. Therefore, the initial state will gradually lose its coherence. Moreover, this incoherent dynamical behavior is governed by the master equation of isotropic depolarization

8 $$\begin{aligned} \frac{\partial }{\partial t}{\overline{\rho }}(t)=\frac{\gamma (t)}{2}\sum _{j=1}^3\left[ {\hat{\sigma }}_j{\overline{\rho }}(t){\hat{\sigma }}_j-{\overline{\rho }}(t)\right] , \end{aligned}$$

with decay rate $\gamma (t)=-{\dot{w}}(t)/2w(t)$; namely, the three Pauli decay channels share identical decay rate. The derivation of the master equation from Eq. (5) is outlined in Ref.^23,32–34^. Additionally, the decohering dynamics of this isotropic depolarization can also be well-understood by the purity

9 $$\begin{aligned} \mathrm {Tr}[{\overline{\rho }}^2(t)]=w^2(t)\left[ \mathrm {Tr}\left( \rho _0^2\right) -\frac{1}{2}\right] +\frac{1}{2}. \end{aligned}$$

It is worthwhile to note that, the spherical symmetry qualitatively reproduces the unitarily invariant disorder for qubit^23^, which is studied by means of incorporating the spectral disorder with the Haar measure distributed uniformly over whole solid angular coordinates. However, one of the drawbacks of this approach is the occurrence of double counting of each member Hamiltonian operator. For example, both of the two identical members $\omega {\hat{\sigma }}_z/2$ and $\exp (-i\pi {\hat{\sigma }}_x/2)(-\omega {\hat{\sigma }}_z/2)\exp (i\pi {\hat{\sigma }}_x/2)$ contribute individually to the ensemble-average procedure. On the other hand, the HE in the canonical form rules out this circumstance by considering positive radial coordinate exclusively. Therefore, quantitative differences can be seen when we further take the radial part $P(\omega )$ into account in the following. To further clarify this situation and to exemplify the isotropic depolarization, we will carry out several types of distribution functions. As the solid angular part $\Theta (\theta ,\phi )$ has been specified according to the symmetry, we will characterize the distribution functions with the radial part $P(\omega )$.

As a comparative study, we consider the Gaussian radial function defined as

10 $$\begin{aligned} P_\mathrm {G}(\omega )= \frac{1}{\omega _\mathrm {c}^3}\sqrt{\frac{2}{\pi }}e^{-\frac{\omega ^2}{2\omega _\mathrm {c}^2}} \end{aligned}$$

for $\omega \ge 0$. Note that the standard deviation $\omega _\mathrm {c}$ controls the width of the distribution and therefore plays similar role of cutoff frequency. The functional form is specified according to the normalization condition $\int _0^\infty P_\mathrm {G}(\omega )\omega ^2d\omega =1$ and therefore the coefficient is slightly different from the usual one. For the Gaussian radial function $P_\mathrm {G}(\omega )$, one can analytically evaluate

11 $$\begin{aligned} \langle \cos \omega t\rangle _{P_\mathrm {G}}=\int _0^\infty \cos \omega t P_\mathrm {G}(\omega )\omega ^2d\omega =e^{-\frac{(\omega _\mathrm {c}t)^2}{2}}\left[ 1-(\omega _\mathrm {c}t)^2\right] . \end{aligned}$$

This allows us to evaluate the mixing weight $w_\mathrm {G}(t)=(2\langle \cos \omega t\rangle _{P_\mathrm {G}}+1)/3$ and the decay rate in Eq. (8):

12 $$\begin{aligned} \gamma _\mathrm {G}(t)=\omega _\mathrm {c}\frac{\omega _\mathrm {c}t\left[ 3-(\omega _\mathrm {c}t)^2\right] }{2\left[ 1-(\omega _\mathrm {c}t)^2\right] +e^{\left[ (\omega _\mathrm {c}t)^2/2\right] }}. \end{aligned}$$

If compared with the one obtained by the unitarily invariant disorder for qubit^23^, we can find that Eq. (12) is lesser by a factor 2, indicating that the HE in the canonical form rules out the circumstance of double counting. The numerical results are shown in Fig. 1b. It can be seen that, $\gamma _\mathrm {G}(t)$ is initially increasing, then followed by a sharp descent to negative values, and finally approaching zero asymptotically from below. Additionally, the time evolution of the purity with pure initial state $\mathrm {Tr}[{\overline{\rho }}^2(t)]=\left[ w_\mathrm {G}^2(t)+1\right] /2$ is shown in Fig. 1c. The purity initially decays very approaching the completely mixed state due to the increasing $\gamma _\mathrm {G}(t)$; afterwards, it rises again and saturates to the value of $(w_\mathrm {G}^2(t\rightarrow \infty )+1)/2$, indicating that the final state deviates from the completely mixed state. This property can also be observed in the following two examples and will be discussed latter.

Next, we consider an alternative cutoff in exponential form defined as

13 $$\begin{aligned} P_\mathrm {EC}(\omega )=\frac{1}{6\omega _\mathrm {c}^4}\omega e^{-\frac{\omega }{\omega _\mathrm {c}}}, \end{aligned}$$

satisfying the normalization condition $\int _0^\infty P_\mathrm {EC}(\omega )\omega ^2d\omega =1$. $\omega _\mathrm {c}$ is the cutoff value. With this exponential cutoff, we have

14 $$\begin{aligned} \langle \cos \omega t\rangle _{P_\mathrm {EC}}=\int _0^\infty \cos \omega t P_\mathrm {EC}(\omega )\omega ^2d\omega =\frac{1-6(\omega _\mathrm {c}t)^2+(\omega _\mathrm {c}t)^4}{\left[ 1+(\omega _\mathrm {c}t)^2\right] ^4}, \end{aligned}$$

the mixing weight $w_\mathrm {EC}(t)=(2\langle \cos \omega t\rangle _{P_\mathrm {EC}}+1)/3$, and the decay rate in Eq. (8):

15 $$\begin{aligned} \gamma _\mathrm {EC}(t)=4\omega _\mathrm {c}^2t\frac{\left[ 3-(\omega _\mathrm {c}t)^2\right] \left[ 1+(\omega _\mathrm {c}t)^2\right] +2\left[ 1-6(\omega _\mathrm {c}t)^2+(\omega _\mathrm {c}t)^4\right] }{2\left[ 1-6(\omega _\mathrm {c}t)^2+(\omega _\mathrm {c}t)^4\right] \left[ 1+(\omega _\mathrm {c}t)^2\right] +\left[ 1+(\omega _\mathrm {c}t)^2\right] ^5}. \end{aligned}$$

From Fig. 1b, $\gamma _\mathrm {EC}(t)$ exhibits a similar behavior to $\gamma _\mathrm {G}(t)$, but even sharper oscillation. Consequently, this is also the case for the purity $\mathrm {Tr}[{\overline{\rho }}^2(t)]=\left[ w_\mathrm {EC}^2(t)+1\right] /2$ shown in Fig. 1c. This is due to the fact that $P_\mathrm {EC}(\omega )$ possesses a long wing over high $\omega$ domain, leading to a rapid spreading of random unitary rotation and shorter coherence time.

As a third example, we consider a radial function of different type. The reciprocal square

16 $$\begin{aligned} P_\mathrm {RS}(\omega )=\frac{1}{\omega _\mathrm {c}\omega ^2}, \end{aligned}$$

for $\omega \in [0,\omega _\mathrm {c}]$, is defined on a finite interval rather than infinite length. Again the functional form is specified by the normalization condition $\int _0^{\omega _\mathrm {c}}P_\mathrm {RS}(\omega )\omega ^2d\omega =1$. Then the radial expectation

17 $$\begin{aligned} \langle \cos \omega t\rangle _{P_\mathrm {RS}}=\int _0^{\omega _\mathrm {c}} \cos \omega t P_\mathrm {RS}(\omega )\omega ^2d\omega =\frac{\sin \omega _\mathrm {c}t}{\omega _\mathrm {c}t} \end{aligned}$$

leads to the mixing weight $w_\mathrm {RS}(t)=(2\langle \cos \omega t\rangle _{P_\mathrm {RS}}+1)/3$ and the decay rate in Eq. (8):

18 $$\begin{aligned} \gamma _\mathrm {RS}(t)=\omega _\mathrm {c}\frac{\sin \omega _\mathrm {c}t-(\omega _\mathrm {c}t)\cos \omega _\mathrm {c}t}{2(\omega _\mathrm {c}t)\sin \omega _\mathrm {c}t+(\omega _\mathrm {c}t)^2}. \end{aligned}$$

Due to the finite domain of $P_\mathrm {RS}(\omega )$, the decay rate $\gamma _\mathrm {RS}(t)$ exhibits a long oscillating tail as shown in Fig. 1b. This behavior is very different from $\gamma _\mathrm {G}(t)$ and $\gamma _\mathrm {EC}(t)$, and renders the purity $\mathrm {Tr}[{\overline{\rho }}^2(t)]=\left[ w_\mathrm {RS}^2(t)+1\right] /2$ oscillating as well after the initial descent.

It is evident from the above three examples that, whenever the decay rates $\gamma (t)$ go down to negative values, there is a revival of the purity $\mathrm {Tr}[{\overline{\rho }}^2(t)]$ in the time period of negative decay rate. We can even clearly observe this phenomenon from the long oscillating tails in the reciprocal square example. This can be explained as a typical transition between indivisibility and full divisibility^34–37^ of the type of RU^38,39^.

On the other hand, although the dynamics is governed by the master equation (8) of isotropic depolarization, the final state is generically not the completely mixed state. This can be realized by observing that the purity saturates to a value of 5/9, rather than 1/2 in Fig. 1c. This does not imply the violation of unitality of the ensemble-averaged dynamics under canonical HE. Since $\langle \cos \omega t\rangle _{\omega ^3/3}\rightarrow 0$ when $t\rightarrow \infty$ in these examples, we have the steady mixing weight $w(t\rightarrow \infty )=1/3$ and, consequently, the final state ${\overline{\rho }}(\infty )=\left[ 2({\widehat{I}}/2)+\rho _0\right] /3$, a constant mixture between the completely mixed state and the initial state $\rho _0$.

We have shown that the spherically symmetric probability distribution qualitatively reproduces the unitarily invariant disorder for qubit and leads to the master equation of isotropic depolarization with identical decay rate of the three Pauli decay channels. To demonstrate the versatility of the canonical HE in characterizing the incoherent dynamics beyond isotropic depolarization, we will further reduce the degree of symmetry and explore its effects on the incoherent dynamical behaviors.

## Simultaneous azimuthal and reflectional symmetries

To release the spherical symmetry, we consider the HE exhibiting simultaneously the azimuthal symmetry and the reflectional symmetry about the *x*-*y* plane. Specifically, we will consider the two examples of unbalanced regimes; namely the bagel-shaped $\Theta (\theta ,\phi )=\pi ^{-2}\sin \theta$ and the dumbbell-shaped $\Theta (\theta ,\phi )=(3/4\pi )\cos ^2\theta$. Under these symmetries, it is straightforward to see that the 1st directional moments vanish again, $\langle n_j\rangle _\Theta =0$, and the 2nd *x*- and *y*-directional moments are equal, $\langle n_x^2\rangle _\Theta =\langle n_y^2\rangle _\Theta$. However, in contrast to the case of spherical symmetry, they may not necessarily equal to the 2nd *z*-directional moment $\langle n_z^2\rangle _\Theta$; meanwhile, without loss of generality, we assume that the diagonal condition of Eq. (4) is still hold, i.e., $\langle n_j n_k\rangle _\Theta =0$ for $j\ne k$.

After determining the directional moments according to the symmetries, the action of ensemble-averaged dynamical linear map in Eq. (5) is significantly simplified as

19 $$\begin{aligned} \left\{ \begin{array}{l} {\mathscr {E}}_t\{{\widehat{I}}\}={\widehat{I}} \\ {\mathscr {E}}_t\{{\hat{\sigma }}_j\}=f_j(t){\hat{\sigma }}_j , j=1,2,3 \end{array}\right., \end{aligned}$$

Since the spherical symmetry is no longer hold, the actions of ${\mathscr {E}}_t$ on the three generators are different. Consequently, the incoherent dynamical behavior is governed by the master equation of anisotropic depolarization

20 $$\begin{aligned} \frac{\partial }{\partial t}{\overline{\rho }}(t)=\sum _{j=1}^3\frac{\gamma _j(t)}{2}\left[ {\hat{\sigma }}_j{\overline{\rho }}(t){\hat{\sigma }}_j-{\overline{\rho }}(t)\right] , \end{aligned}$$

with decay rates $\gamma _j(t)=\left[ {\dot{f}}_j(t)/2f_j(t)\right] -\sum _{k\ne j}{\dot{f}}_k(t)/2f_k(t)$ associated to the corresponding Pauli decay channels. Moreover, due to the azimuthal symmetry, we have $\langle n_x^2\rangle _\Theta =\langle n_y^2\rangle _\Theta$, $f_x(t)=f_y(t)$, and, consequently, $\gamma _x(t)=\gamma _y(t)=-{\dot{f}}_z(t)/2f_z(t)$. Each decay rate can be considered as a competition between $\left[ {\dot{f}}_j(t)/2f_j(t)\right]$’s, which are determined by the 2nd directional moments. Accordingly, we will consider the two unbalanced regimes, $\langle n_x^2\rangle _\Theta =\langle n_y^2\rangle _\Theta>\xi /3>\langle n_z^2\rangle _\Theta$ and $\langle n_x^2\rangle _\Theta =\langle n_y^2\rangle _\Theta<\xi /3<\langle n_z^2\rangle _\Theta$. Moreover, the profile of purity now depends on the initial state $\rho _0=({\widehat{I}}+\vec {\rho }_0\cdot {\hat{\sigma }})/2$:

21 $$\begin{aligned} \mathrm {Tr}[{\overline{\rho }}^2(t)]=\frac{1}{2}\left[ 1+|\vec {\rho }(t)|^2\right] =\frac{1}{2}\left[ 1+\sum _{j=1}^3|\rho _{0,j}f_j(t)|^2\right] . \end{aligned}$$

### Bagel-shaped solid angular function

**Figure 2:**
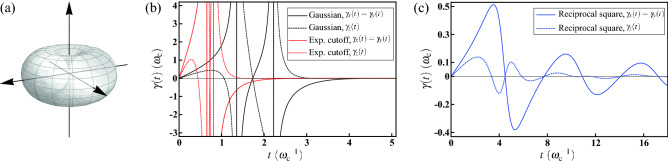
Decoherence under the HE with bagel-shaped geometry. (**a**) Visualization of the solid angular part $\Theta (\theta ,\phi )=\pi ^{-2}\sin \theta$ of the probability distribution. The bagel-shaped geometry exhibits the azimuthal and reflectional symmetries simultaneously; meanwhile, this leads to the regime $\langle n_x^2\rangle _\Theta =\langle n_y^2\rangle _\Theta>1/3>\langle n_z^2\rangle _\Theta$. (**b**) The decay rates $\gamma _x(t)$ (solid curves) and $\gamma _z(t)$ (dashed curves) for the Gaussian (black) and the exponential cutoff (red) radial functions. Each $\gamma _x(t)$ exhibits two singularities and finally approaches zero asymptotically. (**c**) The decay rates $\gamma _x(t)$ (solid curves) and $\gamma _z(t)$ (dashed curves) for the reciprocal square radial function. $\gamma _x(t)$ shows a similar oscillating behavior; while $\gamma _z(t)$ possesses more zeros. Additionally, the amplitude of $\gamma _x(t)$ is larger than $\gamma _z(t)$, reflecting the relationship $\langle n_x^2\rangle _\Theta >\langle n_z^2\rangle _\Theta$.

We first show the former by considering the case of a bagel-shaped solid angular part with $\Theta (\theta ,\phi )=\pi ^{-2}\sin \theta$. Its visualization is shown in Fig. 2a, from which it is obvious that $\Theta (\theta ,\phi )$ exhibits simultaneously the azimuthal symmetry and the reflectional symmetry about the *x*-*y* plane. With the specified functional form, we can explicitly compute $\langle n_x^2\rangle _\Theta =\langle n_y^2\rangle _\Theta =3/8$, $\langle n_z^2\rangle _\Theta =1/4$, and $\xi =\int \Theta (\theta ,\phi )d\Omega =1$, satisfying the required relationship $\langle n_x^2\rangle _\Theta =\langle n_y^2\rangle _\Theta>1/3>\langle n_z^2\rangle _\Theta$. In fact, this relationship can also be inferred from the visualization of $\Theta (\theta ,\phi )$ before explicit computations.

**Figure 3:**
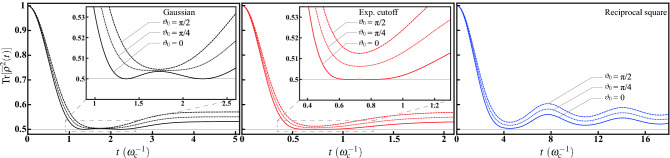
The purities $\mathrm {Tr}[{\overline{\rho }}^2(t)]$ for initial state $\vec {\rho }_0=(\sin \vartheta _0,0,\cos \vartheta _0)$ with $\vartheta _0=0$ (solid curves), $\pi /4$ (dotted curves), and $\pi /2$ (dashed curves), respectively. For $\vartheta _0=0$, strong relation can be observed between the purity and $\gamma _x(t)$ in Fig. 2. Positive (negative) $\gamma _x(t)$ results in lowering (rising) purity, respectively; and the singularity of $\gamma _x(t)$ leads to a full die-out of purity followed by a revival. While the case of $\vartheta _0=\pi /2$ is more involved. The profile of purity is a result of the competition between $\gamma _x(t)$ and $\gamma _z(t)$. Under the regime $\langle n_x^2\rangle _\Theta >\langle n_z^2\rangle _\Theta$, the singular effects of $\gamma _z(t)$ are quenched by $\gamma _x(t)$ and therefore the purity is always finite.

Now we revisit the three radial functions, the Gaussian $P_\mathrm {G}(\omega )$ (10), the exponential cutoff $P_\mathrm {EC}(\omega )$ (13), and the reciprocal square $P_\mathrm {RS}(\omega )$ (16). We first verify that $\int _0^\infty P(\omega )\omega ^2 d\omega =\int \Theta (\theta ,\phi )d\Omega =1$ guarantees the normalization condition for $p(\omega ,\theta ,\phi )$, and the radial expectation $\langle \cos \omega t\rangle _P$ with respect to the three radial functions have been shown in Eqs. (11), (14), and (17), respectively. Then the incoherent dynamical behavior can be fully understood and the decay rates $\gamma _j(t)$ in the master equation (20) can also be computed explicitly. The analytical expressions of $\gamma _j(t)$ are given in Methods. We show the numerical results with respect to the radial functions in Fig. 2.

Figure 2b shows $\gamma _x(t)$ (solid curves) and $\gamma _z(t)$ (dashed curves) for the Gaussian (black) and the exponential cutoff (red) radial functions. Note that, each of the sharp descents of $\gamma _x(t)$ for the Gaussian and the exponential cutoff under spherical symmetry (cf. Fig. 1b) now splits into two prominent singularities under lower symmetry. This renders the behavior of $\gamma _z(t)=-\left[ {\dot{f}}_x(t)/f_x(t)\right] -\gamma _x(t)$ singular as well. Finally, they again approach zero asymptotically. The results for the reciprocal square radial function are shown in Fig. 2c. In contrast to the former, both $\gamma _x(t)$ and $\gamma _z(t)$ are regular. It can be seen that $\gamma _x(t)$ shows similar temporal behavior to the one under spherical symmetry (cf. Fig. 1b), whereas $\gamma _z(t)$ possesses more zeros. Additionally, it is interesting to note that the amplitude of $\gamma _x(t)$ is larger than $\gamma _z(t)$, reminiscent of the relationship $\langle n_x^2\rangle _\Theta >\langle n_z^2\rangle _\Theta$ we are considered. Similar analogy can be observed in the latter example. In fact, this analogy provides some insights into the effects of symmetry breaking on the decay rates, and will be discussed latter.

The numerical results of purity are shown in Fig. 3 for initial state $\vec {\rho }_0=(\sin \vartheta _0,0,\cos \vartheta _0)$ with $\vartheta _0=0$ (solid curves), $\pi /4$ (dotted curves), and $\pi /2$ (dashed curves), respectively. For $\vartheta _0=0$, the time evolution is solely determined by $f_z(t)$ [cf. Eq. (19)], which in turn fully determines $\gamma _x(t)$ and $\gamma _y(t)$. Therefore, the profile of purity completely reflects the behavior of $\gamma _x(t)$ in Fig. 2. It is obvious that positive (negative) $\gamma _x(t)$ results in lowering (rising) purity, respectively; meanwhile, the singularity of $\gamma _x(t)$ leads to a full die-out of purity followed by a revival. On the other hand, it is more involved for $\vartheta _0=\pi /2$. Now $f_x(t)$ dominates the time evolution. This leads to a competition between $\gamma _x(t)$ and $\gamma _z(t)$. The singular effects of $\gamma _z(t)$ are quenched by $\gamma _x(t)$ due to the regime $\langle n_x^2\rangle _\Theta >\langle n_z^2\rangle _\Theta$ and therefore the purity is always finite.

### Dumbbell-shaped solid angular function

**Figure 4:**
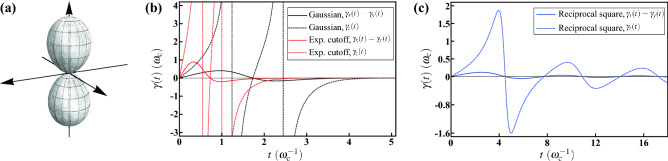
Decoherence under the HE with dumbbell-shaped geometry. (**a**) Visualization of the solid angular part $\Theta (\theta ,\phi )=(3/4\pi )\cos ^2\theta$ of the probability distribution. The dumbbell-shaped geometry exhibits the azimuthal and reflectional symmetries simultaneously; meanwhile, this leads to the regime $\langle n_x^2\rangle _\Theta =\langle n_y^2\rangle _\Theta<1/3<\langle n_z^2\rangle _\Theta$. (**b**) The decay rates $\gamma _x(t)$ (solid curves) and $\gamma _z(t)$ (dashed curves) for the Gaussian (black) and the exponential cutoff (red) radial functions. $\gamma _x(t)$’s are finite with smaller amplitudes, whereas $\gamma _z(t)$’s are singular. Furthermore, $\gamma _x(t)$ for the exponential cutoff approaches zero asymptotically from above after a mild rising. (**c**) The decay rates $\gamma _x(t)$ (solid curves) and $\gamma _z(t)$ (dashed curves) for the reciprocal square radial function. Both of the decay rates exhibits the same temporal behavior. Finally, in these plots, we can see the analogy between the amplitudes of $\gamma _j(t)$’s and the relationship $\langle n_x^2\rangle _\Theta <\langle n_z^2\rangle _\Theta$.

For the second regime we consider the case of a dumbbell-shaped solid angular part with $\Theta (\theta ,\phi )=(3/4\pi )\cos ^2\theta$. Its visualization is shown in Fig. 4a and clearly satisfies the desired simultaneous symmetries. In this case the 2nd directional moments are $\langle n_x^2\rangle _\Theta =\langle n_y^2\rangle _\Theta =1/5$, $\langle n_z^2\rangle _\Theta =3/5$, and $\xi =\int \Theta (\theta ,\phi )d\Omega =1$, satisfying the required relationship $\langle n_x^2\rangle _\Theta =\langle n_y^2\rangle _\Theta<1/3<\langle n_z^2\rangle _\Theta$. Similarly, we adopt the same radial functions again. The analytic expressions are given in Methods. We show the numerical results in Fig. 4.

Figure  4b shows the results of $\gamma _x(t)$ (solid curves) and $\gamma _z(t)$ (dashed curves) for the Gaussian (black) and the exponential cutoff (red) radial functions. Under this regime $\gamma _x(t)$’s are finite with amplitudes smaller than the ones under spherical symmetry [cf. Fig. 1b], whereas $\gamma _z(t)$’s are still singular, reflecting the relationship $\langle n_x^2\rangle _\Theta <\langle n_z^2\rangle _\Theta$ as well. On the other hand, $\gamma _x(t)$ for the Gaussian exhibits a similar temporal behavior to the one under spherical symmetry; while $\gamma _x(t)$ for exponential cutoff exhibits an additional mild rising from negative and finally approaches zero asymptotically from above. The results for the reciprocal square radial function are shown in Fig. 4c. We can see that both of the decay rates exhibits the same temporal behavior and, consequently, possess the same zeros. Meanwhile, We again see the same analogy between the amplitudes of $\gamma _j(t)$’s and the relationship $\langle n_x^2\rangle _\Theta <\langle n_z^2\rangle _\Theta$.

**Figure 5:**
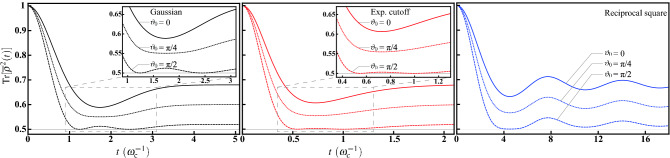
The purities $\mathrm {Tr}[{\overline{\rho }}^2(t)]$ for initial state $\vec {\rho }_0=(\sin \vartheta _0,0,\cos \vartheta _0)$ with $\vartheta _0=0$ (solid curves), $\pi /4$ (dotted curves), and $\pi /2$ (dashed curves), respectively. Aforementioned relation between the purity and $\gamma _j(t)$’s in Fig. 4 can also be observed. However, due to the regime $\langle n_x^2\rangle _\Theta <\langle n_z^2\rangle _\Theta$ considered here, smaller amplitude of $\gamma _x(t)$ implies larger purity for $\vartheta _0=0$. While for the case of $\vartheta _0=\pi /2$, the singular effects of $\gamma _z(t)$ are dominant and therefore lead to a full die-out of purity.

The profile of purity shown in Fig. 5 basically obeys the same logic as that in Fig. 3. However, as here we consider the opposite regime, smaller amplitude of $\gamma _x(t)$ implies larger purity for $\vartheta _0=0$. The singular effects of $\gamma _z(t)$ are now dominant in the competition with $\gamma _x(t)$ and therefore lead to a full die-out of purity for $\vartheta _0=\pi /2$.

It has been pointed out that, for qubit pure dephsing, the dephasing rate is dominated by the variance of the probability distribution within HE^23^. On the other hand, provided the simultaneous symmetries considered here, the 2nd directional moments play the role of variance alone the specified directions. Consequently, the asymmetry goes into play via them, as reflected by the decay rates, and gives rise to the analogy. This point can be understood from the decay rates $\gamma _j(t)$ in Eq. (20), along with $f_j(t)$ in Eq. (6). Furthermore, according to the symmetries under consideration, we have $\langle n_x^2\rangle _\Theta =\langle n_y^2\rangle _\Theta \ne \langle n_z^2\rangle _\Theta$. This implies these two regimes and demonstrates striking difference in the dynamical behavior. In the following, we will further reduce the symmetry by considering the azimuthal symmetry exclusively and explore the effects of 1st directional moment.

## Azimuthal symmetry

**Figure 6:**
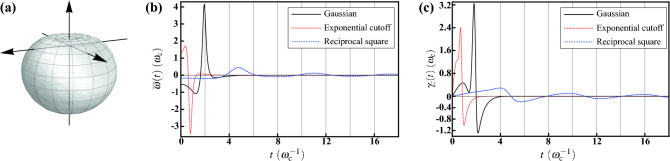
Decoherence under the HE with the geometry of 3D cardioid. (**a**) Visualization of the solid angular part $\Theta (\theta ,\phi )=(1-\cos \theta )/4\pi$ of the probability distribution. This geometry exhibits the azimuthal symmetry exclusively; meanwhile, this leads to the balanced regime $\langle n_x^2\rangle _\Theta =\langle n_y^2\rangle _\Theta =1/3=\langle n_z^2\rangle _\Theta$. (**b**) The averaged level spacing ${\overline{\omega }}(t)$ for the Gaussian (black solid curve), the exponential cutoff (red dotted curve), and the reciprocal square (blue dashed cruve) radial functions. Its presence is a result of the lack of the reflectional symmetry about the *x*-*y* plane. (**c**) The decay rate $\gamma _z(t)$ for the Gaussian (black solid curve), the exponential cutoff (red dotted curve), and the reciprocal square (blue dashed cruve) radial functions. The line shape is similar to the one under spherical symmetry appended by an additional drop before the peaking value, which is a result of the asymmetry.

In the presence of the azimuthal symmetry, it has been shown in the previous examples that the 1st *x*- and *y*-directional moments vanish, $\langle n_x\rangle _\Theta =\langle n_y\rangle _\Theta =0$, and the 2nd *x*- and *y*-directional moments are equal, $\langle n_x^2\rangle _\Theta =\langle n_y^2\rangle _\Theta$. Crucially, in the absence of reflectional symmetry about the *x*-*y* plane, the 1st *z*-directional moment is typically non-vanishing, $\langle n_z\rangle _\Theta \ne 0$. For example, we will consider a solid angular part of three-dimensional cardioid with $\Theta (\theta ,\phi )=(1-\cos \theta )/4\pi$. Its visualization is shown in Fig. 6a.

According to the directional moments determined by the symmetry under consideration, the action of ensemble-averaged dynamical linear map in Eq. (5) can be explicitly written as

22 $$\begin{aligned} \left\{ \begin{array}{l} {\mathscr {E}}_t\{{\widehat{I}}\}={\widehat{I}} \\ {\mathscr {E}}_t\{{\hat{\sigma }}_x\}=f_x(t){\hat{\sigma }}_x+\langle \sin \omega t\rangle _P\langle n_z\rangle _\Theta {\hat{\sigma }}_y\\ {\mathscr {E}}_t\{{\hat{\sigma }}_y\}=f_y(t){\hat{\sigma }}_y-\langle \sin \omega t\rangle _P\langle n_z\rangle _\Theta {\hat{\sigma }}_x\\ {\mathscr {E}}_t\{{\hat{\sigma }}_z\}=f_z(t){\hat{\sigma }}_z \end{array}\right. , \end{aligned}$$

In the presence of $\langle n_z\rangle _\Theta$, we have one more additional radial expectation $\langle \sin \omega t\rangle _P$; meanwhile, the incoherent dynamical behavior is governed by the master equation

23 $$\begin{aligned} \frac{\partial }{\partial t}{\overline{\rho }}(t)=-i\left[ \frac{{\overline{\omega }}(t)}{2}{\hat{\sigma }}_z,{\overline{\rho }}(t)\right] +\sum _{j=1}^3\frac{\gamma _j(t)}{2}\left[ {\hat{\sigma }}_j{\overline{\rho }}(t){\hat{\sigma }}_j-{\overline{\rho }}(t)\right] . \end{aligned}$$

In deriving the above master equation, we have used the facts that $\langle n_x^2\rangle _\Theta =\langle n_y^2\rangle _\Theta$ and $f_x(t)=f_y(t)$; therefore, $\gamma _x(t)=\gamma _y(t)=-{\dot{f}}_z(t)/2f_z(t)$. Note that the most prominent difference of Eq. (23) from the previous examples is the presence of an effective level spacing ${\overline{\omega }}(t)=\langle n_z\rangle _\Theta \left[ f_x(t)(\frac{d}{dt}\langle \sin \omega t\rangle _P)-{\dot{f}}_x(t)\langle \sin \omega t\rangle _P\right] /D(t)$ with $D(t)=f^2_x(t)+\langle n_z\rangle _\Theta ^2\langle \sin \omega t\rangle _P^2$. Additionally, the decay rate $\gamma _z(t)=-\left[ f_x(t){\dot{f}}_x(t)/D(t)\right] -\gamma _x(t)-\left[ \langle n_z\rangle _\Theta ^2\langle \sin \omega t\rangle _P(\frac{d}{dt}\langle \sin \omega t\rangle _P)/D(t)\right]$ is also altered in the presence of finite $\langle n_z\rangle _\Theta$. Both of ${\overline{\omega }}(t)$ and the variation of $\gamma _z(t)$ are results of the lack of the reflectional symmetry about the *x*-*y* plane.

Instead of the unbalanced regimes considered in the two previous examples, now we demonstrate a balanced one with $\langle n_x^2\rangle _\Theta =\langle n_y^2\rangle _\Theta =1/3=\langle n_z^2\rangle _\Theta$ to simplify the complexity. We consider a solid angular part of three-dimensional cardioid with $\Theta (\theta ,\phi )=(1-\cos \theta )/4\pi$. Its visualization is shown in Fig. 6a. We can verify that the desired balanced regime is satisfied, and compute $\langle n_x\rangle _\Theta =\langle n_y\rangle _\Theta =0$ and $\langle n_z\rangle _\Theta =-1/3$. With the same radial functions, we can determine the effective level spacing ${\overline{\omega }}(t)$ and the decay rate $\gamma _j(t)$ in the master equation (23). In addition to $\langle \cos \omega t\rangle _P$, now we need one more radial expectation $\langle \sin \omega t\rangle _P$. The analytical expresstions with respect to the three radial functions are given in Methods.

Thanks to the balanced regime under consideration, we find that $\gamma _x(t)=\gamma _y(t)$ and they are exactly the same as those under spherical symmetry; while this is not the case for $\gamma _z(t)$ due to the breaking of the reflectional symmetry. We therefore show the numerical results of ${\overline{\omega }}(t)$ and $\gamma _z(t)$ in Fig. 6b,c, respectively. For the Gaussian radial function (black solid curve), ${\overline{\omega }}(t)$ begins with a negative value. After a shallow drop, a sharp peak is followed, then going down to negative again, and finally approaching zero asymptotically from below. For the exponential cutoff (red dotted), the line shape is similar to that of Gaussian but an overturned one. However, for the reciprocal square (blue dashed curve), ${\overline{\omega }}(t)$ exhibits a long oscillating tail due to the finite domain of $P_\mathrm {RS}(\omega )$. On the other hand, it is interesting to note that, the overall line shape of $\gamma _z(t)$ is similar to the one under spherical symmetry, but an additional drop before reaching the peaking value. The similarity is a consequence of the balanced regime, according to the aforementioned analogy. However, the asymmetry is also influential by perturbing $\gamma _z(t)$.

## Simultaneous reflectional symmetries

Finally, we consider a more asymmetric case by reducing the continuous azimuthal symmetry to a discrete one, i.e., the simultaneous reflectional symmetries about the *x*-*z* and *y*-*z* planes. In this case, we can only deduce the vanishment of the 1st *x*- and *y*-directional moments, $\langle n_x\rangle _\Theta =\langle n_y\rangle _\Theta =0$. The three 2nd directional moments are generically different due to the lack of the azimuthal symmetry.

**Figure 7:**
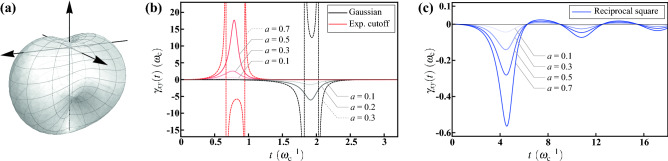
Decoherence under the HE with the geometry of kneaded cardioid. (**a**) Visualization of the solid angular part $\Theta (\theta ,\phi )=(1-\cos \theta )(1+a\cos 2\phi )/4\pi$ with lateral asymmetry $a=0.3$. The azimuthal symmetry is broken due to the $\phi$-dependence, and merely the simultaneous reflectional symmetries about the *x*-*z* and *y*-*z* planes are left. (**b**) The decay rate $\gamma _{xy}(t)$ for the Gaussian (black) and the exponential cutoff (red) radial functions with different lateral asymmetry *a*. As $\gamma _{xy}(t)$ is caused by the azimuthal symmetry breaking, it is gradually vanishing when *a* is decreasing and, contrarily, becomes more prominent for a more asymmetric geometry. As indicated by the dashed curves, the negative peaks even split into two singularities at $a=0.3$ and 0.7 for Gaussian and exponential cutoff radial functions, respectively. (**c**) The decay rate $\gamma _{xy}(t)$ for the reciprocal square radial function with different lateral asymmetry *a*. Similar tendency toward vanishment with decreasing *a* can also be seen. However, for the reciprocal square, $\gamma _{xy}(t)$ do not exhibit singularity even if under large asymmetry.

Given the vanishing 1st *x*- and *y*-directional moments, the action of ensemble-averaged dynamical linear map in Eq. (5) is formally the same as Eq. (22) under azimuthal symmetry. Whereas the corresponding master equation

24 $$\begin{aligned} \frac{\partial }{\partial t}{\overline{\rho }}(t)= & {} -i\left[ \frac{{\overline{\omega }}(t)}{2}{\hat{\sigma }}_z,{\overline{\rho }}(t)\right] +\sum _{j=1}^3\frac{\gamma _j(t)}{2}\left[ {\hat{\sigma }}_j{\overline{\rho }}(t){\hat{\sigma }}_j-{\overline{\rho }}(t)\right] \nonumber \\&+\frac{\gamma _{xy}(t)}{2}\left[ {\hat{\sigma }}_x{\overline{\rho }}(t){\hat{\sigma }}_y-\frac{1}{2}\left\{ {\hat{\sigma }}_y{\hat{\sigma }}_x,{\overline{\rho }}(t)\right\} \right] +\frac{\gamma _{yx}(t)}{2}\left[ {\hat{\sigma }}_y{\overline{\rho }}(t){\hat{\sigma }}_x-\frac{1}{2}\left\{ {\hat{\sigma }}_x{\hat{\sigma }}_y,{\overline{\rho }}(t)\right\} \right] \end{aligned}$$

possesses two more decay channels with the off-diagonal decay rates

25 $$\begin{aligned} \gamma _{xy}(t)=\gamma _{yx}(t)=-\frac{\langle n_z\rangle _\Theta [f_x(t)-f_y(t)]\frac{d}{dt}\langle \sin \omega t\rangle _P}{2D(t)} +\frac{\langle n_z\rangle _\Theta [{\dot{f}}_x(t)-{\dot{f}}_y(t)]\langle \sin \omega t\rangle _P}{2D(t)}, \end{aligned}$$

where $D(t)=f_x(t)f_y(t)+\langle n_z\rangle _\Theta ^2\langle \sin \omega t\rangle _P^2$. It is clear that their presences are a result of the azimuthal symmetry breaking. As this is the lowest degree of symmetry considered here, Eq. (24) is the most general master equation we have demonstrated. It can be reduced to all the previous ones if the corresponding symmetries are recovered.

To exemplify this case of low symmetry, we consider a solid angular part of kneaded cardioid with $\Theta (\theta ,\phi )=(1-\cos \theta )(1+a\cos 2\phi )/4\pi$, where $0\le a\le 1$ describes the degree of lateral asymmetry of the geometry. Its visualization is shown in Fig. 7a with $a=0.3$. Due to the $\phi$-dependence, the geometry appears to be subject to stress along the *y*-axis, and then expanding along the *x*-axis. Therefore the azimuthal symmetry is broken and merely the simultaneous reflectional symmetries are left. For this $\Theta (\theta ,\phi )$, we have $\langle n_z\rangle _\Theta =-1/3$, $\langle n_x^2 \rangle _\Theta =(2+a)/6$, $\langle n_y^2 \rangle _\Theta =(2-a)/6$, and $\langle n_z^2 \rangle _\Theta =1/3$. Along with the radial expectations $\langle \cos \omega t\rangle _P$ shown in Eqs. (11), (14), and (17), as well as $\langle \sin \omega t\rangle _P$ in Methods, the numerical results of $\gamma _{xy}(t)$ (25) are shown in Fig. 7.

The results for the Gaussian radial function are represented in Fig. 7b by black (solid and dashed) curves with varying lateral asymmetry *a*. As expected, $\gamma _{xy}(t)$ is gradually vanishing with decreasing *a* (i.e., more symmetric geometry), reflecting the fact that $\gamma _{xy}(t)$ is a result of azimuthal symmetry breaking. In contrast, $\gamma _{xy}(t)$ becomes more prominent when *a* is large. The negative peak even splits into two singularities at $a=0.3$, as indicated by the black dashed curve. Moreover, the results for the exponential cutoff radial function are represented by red (solid and dashed) curves with varying *a*. It can be seen that the overall tendency is the same as Gaussian but an overturned one. The curves show a positive peak at an earlier time; meanwhile, the exponential-cutoff case shows singularities at a larger value of $a=0.7$, as indicated by the red dashed curve.

On the other hand, the results for the reciprocal square radial function are represented in Fig. 7c by blue curves with varying *a*. The tendency toward vanishment with decreasing *a* can also be seen; whereas, in contrast to the other two radial functions, the deformation of the line shape with *a* is even, without showing singularity, even if *a* is large. Moreover, akin to the other decay rates with the same reciprocal square radial function, $\gamma _{xy}(t)$ also exhibits an oscillating tail, after the negative main peak. Crucially, from these results of Gaussian and reciprocal square radial functions, there is a distinct property the off-diagonal decay rate $\gamma _{xy}(t)$ can be observed as well. To guarantee the complete positivity of the dynamical linear map ${\mathscr {E}}_t$, the short time behavior of all the digonal decay rates $\gamma _j(t)$ associated to the Pauli decay channels is rising at beginning. Any negative values can only emerge after a period of positive values. However, $\gamma _{xy}(t)$ is possible to have a short time behavior of descent to negative at beginning. We stress that this does not imply the violation of complete positivity as the eigenvalues of the Kossakowski matrix ${\mathscr {K}}=[\gamma _{jk}(t)]$ exhibit a short time behavior of rising to positive at beginning.

## Conclusion and discussion

In summary, we have explored the incoherent dynamics of qubits raised from the ensemble average over the canonical HE of structural disorder. Under the variable separation (3) of the probability distribution within the HE into the radial and the solid angular parts, the structural disorder can be characterized in accordance with the degree of symmetry of the solid angular geometry. The effects of asymmetry are particularly manifest via the corresponding master equation in the Lindblad form.

In this work we have considered three radial functions; namely, the Gaussian, the exponential cutoff, and the reciprocal square. We first show the most symmetric case of spherical symmetry, leading to the master equation of isotropic depolarization. It is worthwhile to note that the canonical HE of spherical symmetry not only reproduces the results of unitarily invariant disorder for qubit^23^, but also resolves the issue of double counting. Further versatility of the canonical HE in describing various types of incoherent dynamics beyond isotropic depolarization is revealed by considering asymmetric cases. In addition to the spherical symmetry, we have also demonstrated three lower degrees of symmetry. We can observe the effects caused by the symmetry breaking of different types via the master equation in the Lindblad form, including the singularities of the decay rates, the effective level spacing, and the off-diagonal decay rates. Generally speaking, the more asymmetric the geometry is, the more terms emerge.

Notably, the asymmetry goes into play via the 1st and 2nd directional moments, which in turn determine the aforementioned terms in the master equation. The effective level spacing ${\overline{\omega }}(t)$ emerges in the presence of 1st directional moments, which are caused by the reflectional symmetry breaking. They denote the mean values alone the specified directions. While the time dependence of ${\overline{\omega }}(t)$ is determined by the 2nd directional moments and the radial expectations, characterizing the overall asymmetry and the radial disorder, respectively. On the other hand, the behaviors, particularly the time dependence, of the decay rates are also intimately related to the directional moments. This can be easily understood from the analogy between the relationship of the 2nd directional moments (balanced/unbalanced regimes) and the amplitudes of the decay rates. Moreover, the presence of 1st directional moments will further complicate the decay rates. Finally, the highly asymmetric case gives rise to the emergence of off-diagonal decay rates. Interestingly, very similar conclusions have been drawn under the framework of qubit pure dephasing^23^, where it has been shown that ${\overline{\omega }}(t)$ is given by the mean value and the asymmetry of the distribution, while the dephasing rate is given by the variance and the kurtosis.

Finally, it is worthwhile to note that the variable separation (3) can be further released by considering correlated radial and angular coordinates, leading to even more general joint probability distributions $p(\omega ,\theta ,\phi )$. For example, expansion in terms of spherical harmonics $\mathrm {Y}_{l,m}(\theta ,\phi )$ has been demonstrated in Ref.^30^. Furthermore, this problem can also be investigted from the viewpoint of RU, which is highly related to HE. Since the RU representation is a useful tool in the study of open system dynamics^31,38,39^, considerable efforts have been devoted into the investigation of UR decomposition^40–42^. For example, master equation in the same form as Eq. (20) is derived by mixing fluctuating Gaussian noise along different angles^39^.

On the other hand, it is know that any qubit or qutrit phase damping dynamical mpas are RU^43,44^. However, neither the RU-decomposition nor the extreme points for higher dimensional cases are unclear. These problems can in general merely be numerically implemented^43^. Therefore, the canonical HE would not only provide insights into the effects of higher asymmetry beyond the framework of existing RU representation, but also establish a systematic approach to single out the attainable subset, and substantially simplify the problem of RU-decomposition. This underpins the significance of our approach.

## Methods

### Orthogonal transform of the 2nd directional moments

According to the last term of Eq. (2) in the main text, there should be 9 terms in the last summation of Eq. (4):

26 $$\begin{aligned} \sum _{j',k'=1}^3\langle n_{j'}n_{k'} \rangle _\Theta {\hat{\sigma }}_{j'}\rho _0{\hat{\sigma }}_{k'}. \end{aligned}$$

Since the 9 terms of 2nd directional moment $\langle n_{j'}n_{k'} \rangle _\Theta$ forms a symmetric matrix, it can be diagonalized with an appropriate orthogonal matrix ${\hat{u}}=[u_{j'j}]$ such that

27 $$\begin{aligned} \langle n_{j'}n_{k'} \rangle _\Theta =\sum _{j,k=1}^{3}u_{j'j}\delta _{jk}\langle n_{j}^2\rangle _\Theta u_{k'k}. \end{aligned}$$

This corresponds to a basis transformation ${\hat{\sigma }}_j=\sum _{j'=1}^3u_{j'j}{\hat{\sigma }}_{j'}$ and a rotation $n_j=\sum _{j'=1}^3u_{j'j}n_{j'}$ of the directional unit vector. The later is necessary in the first summation of Eq. (4).

### Decoherence under simultaneous azimuthal and reflectional symmetries

As explained in the main text, given the probability distribution $p(\omega ,\theta ,\phi )=P(\omega )\Theta (\theta ,\phi )$ with the solid angular part $\Theta (\theta ,\phi )$ exhibiting simultaneously the azimuthal symmetry and the reflectional symmetry about the *x*-*y* plane, we can compute the decay rates

28 $$\begin{aligned} \left\{ \begin{array}{l} \gamma _x(t)=\gamma _y(t)=-{\dot{f}}_z(t)/2f_z(t)\\ \gamma _z(t)=-\left[ {\dot{f}}_x(t)/f_x(t)\right] +\left[ {\dot{f}}_z(t)/2f_z(t)\right] =-\left[ {\dot{f}}_x(t)/f_x(t)\right] -\gamma _x(t) \end{array}\right. \end{aligned}$$

in the master equation (20) of anisotropic depolarization, where $f_j(t)=\langle \cos \omega t\rangle _P\left( \xi -\langle n_j^2 \rangle _\Theta \right) +\langle n_j^2 \rangle _\Theta /\xi$. In these expressions, we have used the facts that $\langle n_x^2\rangle _\Theta =\langle n_y^2\rangle _\Theta$ and $f_x(t)=f_y(t)$ due to the azimuthal symmetry.

### Decay rates for bagel-shaped geometry

Given the solid angular part $\Theta (\theta ,\phi )=\pi ^{-2}\sin \theta$, we can further compute $\langle n_x^2\rangle _\Theta =\langle n_y^2\rangle _\Theta =3/8$, $\langle n_z^2\rangle _\Theta =1/4$, and $\xi =\int \Theta (\theta ,\phi )d\Omega =1$.

To fully determine the probability distribution $p(\omega ,\theta ,\phi )=P(\omega )\Theta (\theta ,\phi )$, we revisit the three radial functions defined in Eqs. (10), (13), and (16) in the main text. Then the radial expectation $\langle \cos \omega t\rangle _P$ with respect to the three radial functions are shown in Eqs. (11), (14), and (17), respectively. Combining all the necessary information above, we obtain the decay rates for Gaussian $P_\mathrm {G}(\omega )$

29 $$\begin{aligned} \left\{ \begin{array}{l} \gamma _x(t)=\gamma _y(t)=3\omega _\mathrm {c}\frac{\omega _\mathrm {c}t\left[ 3-(\omega _\mathrm {c}t)^2\right] }{6\left[ 1-(\omega _\mathrm {c}t)^2\right] +2\exp \left[ (\omega _\mathrm {c}t)^2/2\right] }\\ \gamma _z(t)=5\omega _\mathrm {c}\frac{\omega _\mathrm {c}t\left[ 3-(\omega _\mathrm {c}t)^2\right] }{5\left[ 1-(\omega _\mathrm {c}t)^2\right] +3\exp \left[ (\omega _\mathrm {c}t)^2/2\right] }-\gamma _x(t) \end{array}\right., \end{aligned}$$

for exponential cutoff $P_\mathrm {EC}(\omega )$

30 $$\begin{aligned} \left\{ \begin{array}{l} \gamma _x(t)=\gamma _y(t)=6\omega _\mathrm {c}^2t\frac{\left[ 3-(\omega _\mathrm {c}t)^2\right] \left[ 1+(\omega _\mathrm {c}t)^2\right] +2\left[ 1-6(\omega _\mathrm {c}t)^2+(\omega _\mathrm {c}t)^4\right] }{3\left[ 1-6(\omega _\mathrm {c}t)^2+(\omega _\mathrm {c}t)^4\right] \left[ 1+(\omega _\mathrm {c}t)^2\right] +\left[ 1+(\omega _\mathrm {c}t)^2\right] ^5}\\ \gamma _z(t)=20\omega _\mathrm {c}^2t\frac{\left[ 3-(\omega _\mathrm {c}t)^2\right] \left[ 1+(\omega _\mathrm {c}t)^2\right] +2\left[ 1-6(\omega _\mathrm {c}t)^2+(\omega _\mathrm {c}t)^4\right] }{5\left[ 1-6(\omega _\mathrm {c}t)^2+(\omega _\mathrm {c}t)^4\right] \left[ 1+(\omega _\mathrm {c}t)^2\right] +3\left[ 1+(\omega _\mathrm {c}t)^2\right] ^5}-\gamma _x(t) \end{array}\right., \end{aligned}$$

and for reciprocal square $P_\mathrm {RS}(\omega )$

31 $$\begin{aligned} \left\{ \begin{array}{l} \gamma _x(t)=\gamma _y(t)=3\omega _\mathrm {c}\frac{\sin \omega _\mathrm {c}t-(\omega _\mathrm {c}t)\cos \omega _\mathrm {c}t}{6(\omega _\mathrm {c}t)\sin \omega _\mathrm {c}t+2(\omega _\mathrm {c}t)^2}\\ \gamma _z(t)=5\omega _\mathrm {c}\frac{\sin \omega _\mathrm {c}t-(\omega _\mathrm {c}t)\cos \omega _\mathrm {c}t}{5(\omega _\mathrm {c}t)\sin \omega _\mathrm {c}t+3(\omega _\mathrm {c}t)^2}-\gamma _x(t) \end{array}\right., \end{aligned}$$

respectively. The numerical results are shown in Fig. 2.

### Decay rates for dumbbell-shaped geometry

Given the solid angular part $\Theta (\theta ,\phi )=(3/4\pi )\cos ^2\theta$, we can further compute $\langle n_x^2\rangle _\Theta =\langle n_y^2\rangle _\Theta =1/5$, $\langle n_z^2\rangle _\Theta =3/5$, and $\xi =\int \Theta (\theta ,\phi )d\Omega =1$. Given the same radial functions again, then we have the same radial expectations $\langle \cos \omega t\rangle _P$ as shown in Eqs. (11), (14), and (17), respectively. We can compute the decay rates for Gaussian $P_\mathrm {G}(\omega )$

32 $$\begin{aligned} \left\{ \begin{array}{l} \gamma _x(t)=\gamma _y(t)=\omega _\mathrm {c}\frac{\omega _\mathrm {c}t\left[ 3-(\omega _\mathrm {c}t)^2\right] }{2\left[ 1-(\omega _\mathrm {c}t)^2\right] +3\exp \left[ (\omega _\mathrm {c}t)^2/2\right] }\\ \gamma _z(t)=4\omega _\mathrm {c}\frac{\omega _\mathrm {c}t\left[ 3-(\omega _\mathrm {c}t)^2\right] }{4\left[ 1-(\omega _\mathrm {c}t)^2\right] +\exp \left[ (\omega _\mathrm {c}t)^2/2\right] }-\gamma _x(t) \end{array}\right., \end{aligned}$$

for exponential cutoff $P_\mathrm {EC}(\omega )$

33 $$\begin{aligned} \left\{ \begin{array}{l} \gamma _x(t)=\gamma _y(t)=4\omega _\mathrm {c}^2t\frac{\left[ 3-(\omega _\mathrm {c}t)^2\right] \left[ 1+(\omega _\mathrm {c}t)^2\right] +2\left[ 1-6(\omega _\mathrm {c}t)^2+(\omega _\mathrm {c}t)^4\right] }{2\left[ 1-6(\omega _\mathrm {c}t)^2+(\omega _\mathrm {c}t)^4\right] \left[ 1+(\omega _\mathrm {c}t)^2\right] +3\left[ 1+(\omega _\mathrm {c}t)^2\right] ^5}\\ \gamma _z(t)=16\omega _\mathrm {c}^2t\frac{\left[ 3-(\omega _\mathrm {c}t)^2\right] \left[ 1+(\omega _\mathrm {c}t)^2\right] +2\left[ 1-6(\omega _\mathrm {c}t)^2+(\omega _\mathrm {c}t)^4\right] }{4\left[ 1-6(\omega _\mathrm {c}t)^2+(\omega _\mathrm {c}t)^4\right] \left[ 1+(\omega _\mathrm {c}t)^2\right] +\left[ 1+(\omega _\mathrm {c}t)^2\right] ^5}-\gamma _x(t) \end{array}\right., \end{aligned}$$

and for reciprocal square $P_\mathrm {RS}(\omega )$

34 $$\begin{aligned} \left\{ \begin{array}{l} \gamma _x(t)=\gamma _y(t)=\omega _\mathrm {c}\frac{\sin \omega _\mathrm {c}t-(\omega _\mathrm {c}t)\cos \omega _\mathrm {c}t}{2(\omega _\mathrm {c}t)\sin \omega _\mathrm {c}t+3(\omega _\mathrm {c}t)^2}\\ \gamma _z(t)=4\omega _\mathrm {c}\frac{\sin \omega _\mathrm {c}t-(\omega _\mathrm {c}t)\cos \omega _\mathrm {c}t}{4(\omega _\mathrm {c}t)\sin \omega _\mathrm {c}t+(\omega _\mathrm {c}t)^2}-\gamma _x(t) \end{array}\right., \end{aligned}$$

respectively. The numerical results are shown in Fig. 4.

### Radial expectations for the three radial functions

In the absence of the reflectional symmetry, we encounter an additional radial expectation $\langle \sin \omega t\rangle _P$ in Eq. (22), which is necessary in determining the effective level spacing and the decay rates. We can compute $\langle \sin \omega t\rangle _P$ analytically for Gaussian $P_\mathrm {G}(\omega )$

35 $$\begin{aligned} \langle \sin \omega t\rangle _{P_\mathrm {G}(\omega )}=\int _0^\infty \sin \omega t P_\mathrm {G}(\omega )\omega ^2d\omega =\sqrt{\frac{2}{\pi }}\omega _\mathrm {c}t+ e^{-\frac{(\omega _\mathrm {c}t)^2}{2}}\left[ 1-(\omega _\mathrm {c}t)^2\right] \mathrm {erfi}\left( \frac{\omega _\mathrm {c}t}{\sqrt{2}}\right) , \end{aligned}$$

for exponential cutoff $P_\mathrm {EC}(\omega )$

36 $$\begin{aligned} \langle \sin \omega t\rangle _{P_\mathrm {EC}}=\int _0^\infty \sin \omega t P_\mathrm {EC}(\omega )\omega ^2d\omega =\frac{-4\omega _\mathrm {c}t+4(\omega _\mathrm {c}t)^3}{\left[ 1+(\omega _\mathrm {c}t)^2\right] ^4}, \end{aligned}$$

and for reciprocal square $P_\mathrm {RS}(\omega )$

37 $$\begin{aligned} \langle \sin \omega t\rangle _{P_\mathrm {RS}}=\int _0^{\omega _\mathrm {c}}\sin \omega t P_\mathrm {RS}(\omega )\omega ^2d\omega =\frac{1-\cos \omega _\mathrm {c}t}{\omega _\mathrm {c}t}, \end{aligned}$$

respectively.
